# Tooth loss and the risk of cognitive decline and dementia: A meta-analysis of cohort studies

**DOI:** 10.3389/fneur.2023.1103052

**Published:** 2023-04-17

**Authors:** Liqing Li, Qi Zhang, Di Yang, Sule Yang, Yulan Zhao, Min Jiang, Xiaofang Wang, Ling Zhao, Qi Liu, Zuxun Lu, Xiaogang Zhou, Yong Gan, Chunmei Wu

**Affiliations:** ^1^Research Center of Health Policy and Innovation, Jiangxi Science and Technology Normal University, Nanchang, Jiangxi, China; ^2^School of Public Health, Tongji Medical College, Huazhong University of Science and Technology, Wuhan, Hubei, China; ^3^Key Laboratory of Prevention and Treatment of Cardiovascular and Cerebrovascular Diseases of Ministry of Education, Gannan Medical University, Ganzhou, Jiangxi, China; ^4^School of Public Health and Health Management, Gannan Medical University, Ganzhou, Jiangxi, China; ^5^School of Economics and Management, East China Jiaotong University, Nanchang, Jiangxi, China

**Keywords:** dementia, cognitive decline, meta-analysis, Alzheimer's disease, tooth loss

## Abstract

**Introduction:**

Epidemiological studies have shown that tooth loss may be associated with an increased risk of cognitive decline and dementia. However, some results do not show a significant association. Therefore, we performed a meta-analysis to evaluate this association.

**Methods:**

Relevant cohort studies were searched in PubMed, Embase, Web of Science (up to May 2022), and the reference lists of retrieved articles. The pooled relative risk (*RR*) and 95% confidence intervals were computed using a random-effects model (*CI*). Heterogeneity was evaluated using the *I*^2^ statistic. Publication bias was evaluated using the Begg's and Egger's tests.

**Results:**

Eighteen cohort studies met the inclusion criteria. Original studies with 356,297 participants with an average follow-up of 8.6 years (ranging from 2 to 20 years) were included in this study. The pooled *RRs* of tooth loss on dementia and cognitive decline were 1.15 (95% *CI*: 1.10–1.20; *P* < 0.01, *I*^2^ = 67.4%) and 1.20 (95% *CI*: 1.14–1.26; *P* = 0.04, *I*^2^ = 42.3%), respectively. The results of the subgroup analysis showed an increased association between tooth loss and Alzheimer's disease (AD) (*RR* = 1.12, 95% *CI*: 1.02–1.23) and vascular dementia (VaD) (*RR* = 1.25, 95% *CI*: 1.06–1.47). The results of the subgroup analysis also showed that pooled RRs varied by geographic location, sex, use of dentures, number of teeth or edentulous status, dental assessment, and follow-up duration. None of the Begg's and Egger's tests or funnel plots showed evidence of publication bias.

**Discussion:**

Tooth loss is associated with a significantly increased risk of cognitive decline and dementia, suggesting that adequate natural teeth are important for cognitive function in older adults. The likely mechanisms mostly suggested include nutrition, inflammation, and neural feedback, especially deficiency of several nutrients like vitamin D.

## 1. Introduction

Dementia, also known as severe neurocognitive disorder, describes a group of disorders characterized by very poor cognitive function that often affects older people (1). The most common forms are AD and vascular dementia (VaD), which are caused by stroke and other cerebrovascular diseases. Cognitive decline is not the only key symptom of dementia; it has been shown that in many patients it may exist long before the diagnosis of dementia (2, 3). Cognitive decline and dementia lead to impaired ability to perform basic activities of daily living, resulting in a tremendous disease burden and economic burden. According to the World Health Organization, the number of dementia patients currently stands at about 55 million and is increasing by nearly 10 million annually, resulting in care costs of more than $1 trillion worldwide (4, 5). Current treatments for dementia have no curative effect, whereas early prevention, diagnosis, and intervention may delay or even prevent disease progression. Although dementia is not an inevitable part of aging, age is clearly the greatest known risk factor. However, knowledge of risk factors for dementia and cognitive decline is currently limited. More attention should be paid to identifying modifiable risk factors.

Tooth loss is another public health problem in older adults worldwide (6). Recently, interest in the relationship between tooth loss and cognitive decline and dementia has increased. Kondo et al. (7) conducted a case-control study in Japan in 1994 to assess lifestyle factors and found that tooth loss was a significant risk factor for AD. Several types of observational studies have also reported that tooth loss is associated with increased prevalence or incidence of dementia (8). However, negative results have been reported (9). Additionally, tooth loss has also been shown to be related to systemic health, such as stroke (10), obesity (11), cardiovascular diseases (12), cancers (13), and mental illnesses (14, 15). This implies complicated mechanisms between tooth loss and health outcomes, including cognitive damage.

Several meta-analyses have also been conducted. In 2016, Shen et al. (16) published a meta-analysis of six cohort studies, three cross-sectional studies, and two case-control studies to examine the association between tooth loss and the incidence of dementia (odds ratio [*OR*] = 1.43; 95% *CI*: 1. 26–1.63). In the same year, a meta-analysis by Cerutti-Kopplin et al. (17) found a greater than 20% increased risk of developing cognitive decline with a hazard ratio (*HR*) of 1.26 (95% *CI*: 1.14–1.40) and dementia (*HR* = 1.22, 95% *CI*: 1.04–1.43) for people with suboptimal dentition (<20 teeth) based on eight cohort studies. Three meta-studies conducted in 2018 showed similar positive results from all types of observational studies (8) or cohort studies, only (18, 19). The five reviews had limitations, including insufficient (8, 16–19) or poor-qualified studies (19), or unrestricted cohort studies (8, 16). Since then, many new cohort studies have emerged looking at the risks of tooth loss to cognitive decline and dementia (20–23). Therefore, we conducted an updated analysis to further investigate the association between tooth loss and risk of cognitive decline and dementia using well-qualified large sample cohort studies that would provide evidence-based recommendations and guidance for a public health response to dementia.

## 2. Materials and methods

### 2.1. Search strategy

This meta-analysis was not registered and carried out in accordance with the checklist of the Meta-analysis Of Observational Studies in Epidemiology (MOOSE) guidelines and not registered (24) Two researchers separately searched the PubMed, Embase, Web of Science, and China National Knowledge Infrastructure (CNKI) databases from their inception to May 2022 for pertinent studies published in any language. We used the following keywords “oral health,” “dental loss,” “tooth loss,” “periodontitis,” “edentulous,” “missing tooth,” “dental care,” “denture,” “oral disease,” or “poor tooth health” and “dementia,” “Alzheimer's disease,” “AD,” “cognitive impairment,” “mild cognitive impairment,” “MCI,” “cognitive decline,” “neurocognitive disorder,” “cognitive disorder,” “memory disorder,” “vascular dementia,” or “VaD” along with “cohort studies,” “follow-up studies,” “prospective studies,” or “longitudinal studies” (see Supplementary material for details). Additionally, all identified pertinent publications' reference lists were examined.

### 2.2. Inclusion and exclusion criteria

Two investigators independently assessed each cohort study's eligibility, and any disagreements were settled by consultation with a third investigator. Studies that met the following criteria were included in this meta-analysis: (1) tooth loss served as the exposure, and dementia and its subtypes or cognitive decline served as the outcome; (2) the study design was a cohort study, including nested case-control studies with a prospective design or a retrospective cohort study; and (3) the study provided *HR* or relative risk (*RR*) with corresponding 95% *CI* for the association between tooth loss and dementia and/or cognitive decline.

Studies were excluded if any of the following conditions were met: (1) the study was not published as full reports, such as comments, communication, conference abstracts, and letters to editors; (2) participants were patients with dementia at baseline; (3) the study design was cross-sectional or case-control; and (4) the study was not *HR*/*RR* or lacked sufficient data to calculate them.

### 2.3. Data extraction

Two researchers independently collected data from the included studies and discussed the discrepancies; then an entire research group reviewed and confirmed the data. The following information was extracted from the included studies: first author(s) name, year of publication, sex, sample, study name, country, type, baseline age, tooth loss, denture wearing, assessment method, outcome and assessment, sample size, follow-up period, and covariates adjusted for in statistical analysis.

### 2.4. Quality assessment

Utilizing three criteria from the Newcastle-Ottawa scale for cohort studies independently, two researchers evaluated the quality assessment of the meta-analysis (25). Scores of 0–3, 4–6, and 7–9 were assigned for low, moderate, and high quality of studies, respectively. By discussing with a third investigator, disagreements on quality assessment were resolved.

### 2.5. Statistical analysis

*RR* is regarded as a typical measure of the relationship between tooth loss and the risk of dementia and cognitive decline. We preferentially pooled multivariable adjusted risk estimates when such estimates were reported. We combined the unadjusted estimates in the absence of an adjusted analysis. For studies that reported outcomes separately for different age groups, education levels, dental conditions, or dementia types, we combined the estimates using a fixed-effects model to obtain an overall estimate that was combined in the primary meta-analysis (26). To examine the source of heterogeneity in the primary outcomes, subgroup analyses were conducted, stratified by geographic location, sex, use of dentures, number of teeth, edentulous status, methods of dental assessment, and duration of follow-up. In a sensitive analysis to assess robustness, we performed a leave-one-out analysis to observe the magnitude of the effect on the pooled *RR* of each study (24).

The *I*^2^ statistic was used to assess the statistical heterogeneity among studies, with cut-off values of 25%, 50%, and 75%, respectively, designating low, moderate, and high levels of heterogeneity (26). Potential publication bias was evaluated with a funnel plot, the Begg's test (27), and the Egger's test. Stata statistical software (version 12.0; College Station, TX, USA) was used to perform all statistical analyses. All tests were two-sided, and a statistically significant result was defined as *P* < 0.05.

### 2.6. Patient and public involvement statement

Patients and public were not involved in this study.

## 3. Results

### 3.1. Literature search

**Figure 1:**
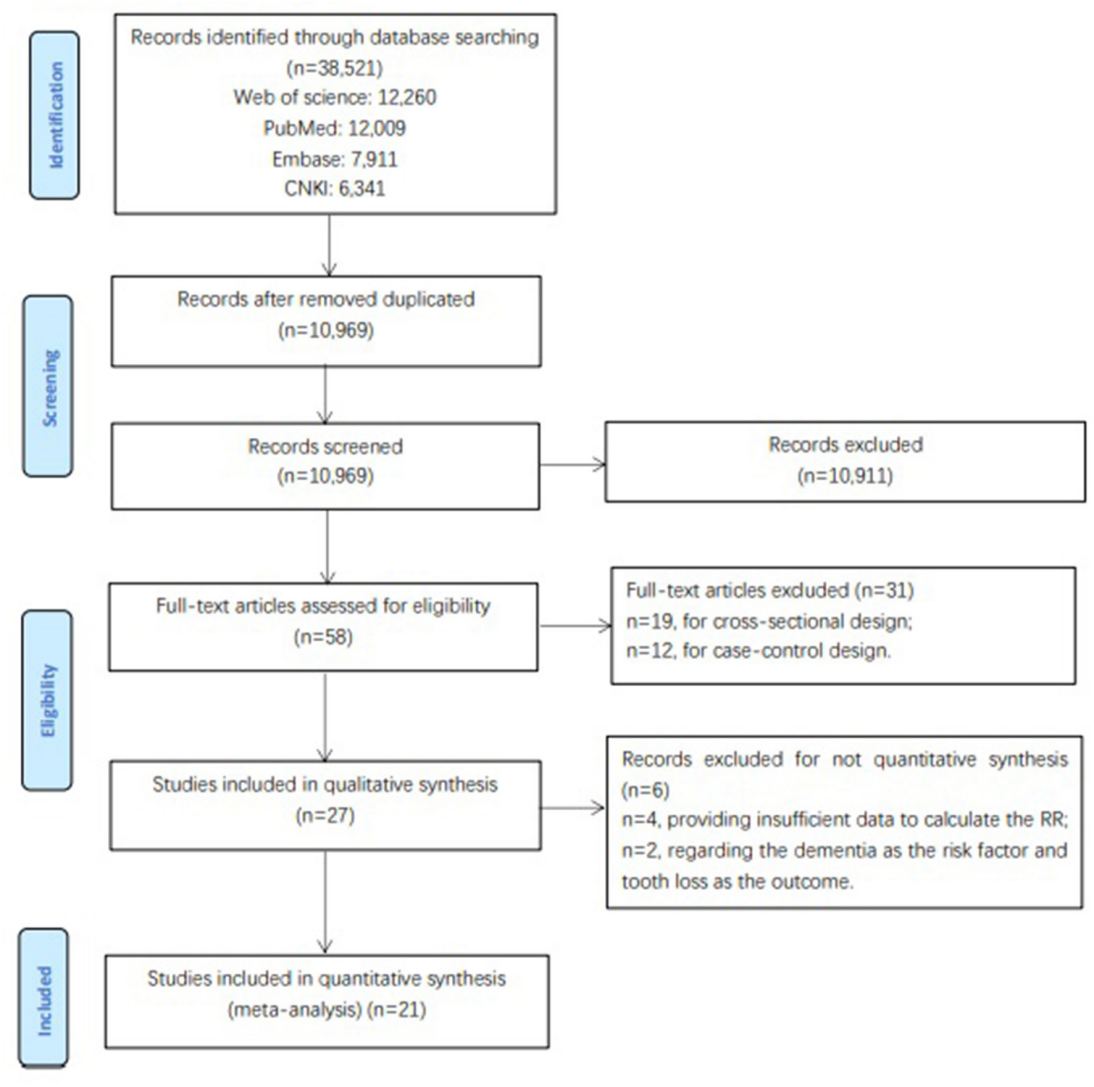
Flow chart of study selection.

Figure 1 illustrates the study selection process. First, 38,521 articles were reviewed. Of these, 27,552 duplicate articles and 10,911 articles that were ineligible for this review after screening based on title and abstract were removed. Of the remaining 58 articles for full-text review, 31 were excluded based on a cross-sectional design (*n* = 19) or a case-control design (*n* = 12). Four studies did not provide sufficient data to calculate, and two studies that had dementia as a risk factor and tooth loss as outcomes were removed. Thus, 21 eligible cohort studies were included in this meta-analysis.

### 3.2. Characteristics of the study

The 21 included studies (9, 22, 23, 25, 28–44) were published between 2001 (28) and 2022 (43, 44), and characteristics of them are shown in Supplementary Table 1. The sample sizes of the cohorts ranged from 133 (30) to 209,806 (22) with a total of 356,297 and the average of 16,967. The length of follow-up duration ranged from 2 (29) to 20 (38) years in our study, with an average of 8.6 years. Among the 14 studies that reported dementia as outcome (9, 22, 23, 29–31, 33–35, 38, 39, 41–43), six studies also reported risk of tooth loss for AD (23, 29, 30, 39, 41, 42), and three studies reported VaD (23, 39, 41), and one reported comorbidity of AD and VaD (23). A various of dementia assessments were applied, including self- or professional-administrated questionnaires or interviews, medical records, and insurance records. A series of tests were also used, and the Mini-mental State Examination (MMSE) was applied in 11 included studies (25, 29, 30, 32, 33, 35–37, 39, 41, 44) as the most frequently used test, while other tests were only used in several studies. Eight included studies reported results with cognitive decline as outcome (25, 28, 32, 35–37, 40, 44), among which one study reported risks of tooth loss for dementia as well (35). Among these studies, ten studies were from Asia (22, 23, 25, 28, 29, 34, 41–44), six studies from North America (30–33, 36, 37), four studies from Europe (33, 38–40), and one study was conducted in 20 countries including Australasia, Asia, Europe and North America (35). Only four studies reported results for male (32, 33, 43), five studies independently reported results for female (30, 33, 39, 43), and the 13 remaining studies (9, 22, 23, 25, 28, 29, 31, 34–38, 40, 41, 44) did not report results by sex.

Supplementary Table 2 shows the results of the quality assessment. The median quality assessment score for the included cohort studies was 8 [range, 5 (31)−9 (22, 25, 28, 29, 41)]. The interobserver agreement (κ) between the two investigators was 0.95.

### 3.3. Quantitative synthesis

#### 3.3.1. Association between tooth loss and dementia

**Figure 2:**
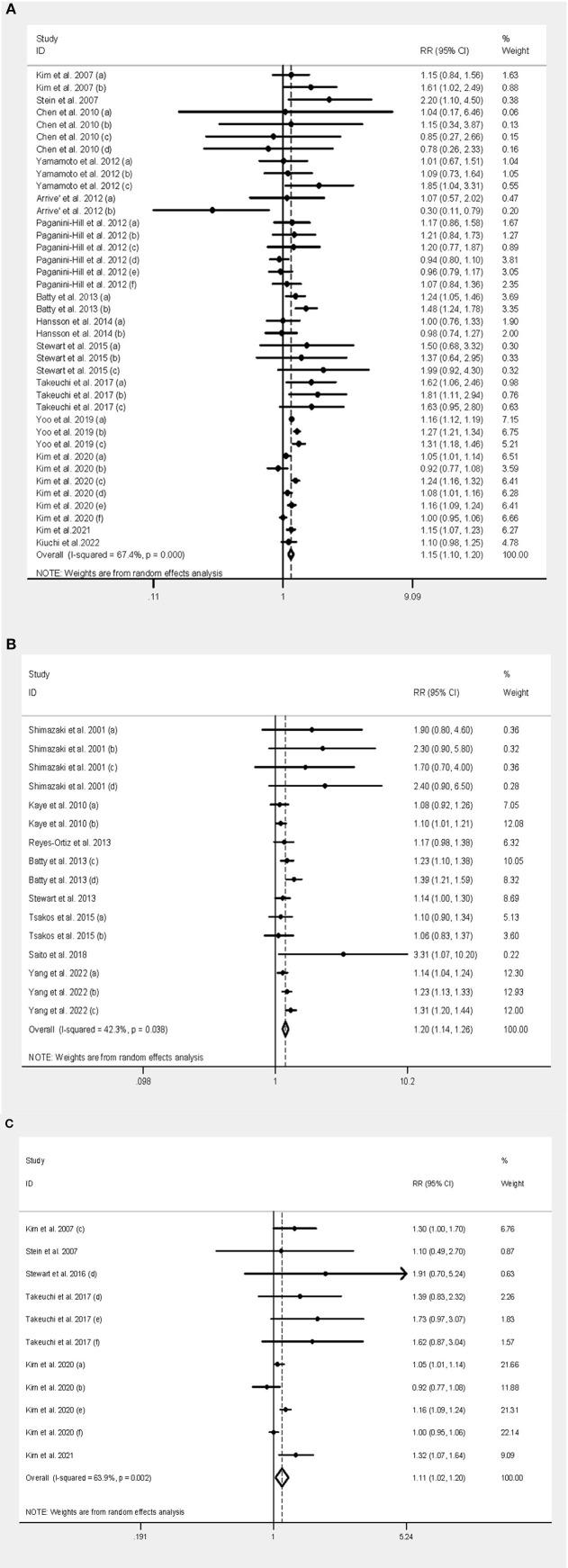
Forest plot of tooth loss and both dementia and cognitive decline. **(A)** Forest plot of tooth loss and dementia. **(B)** Forest plot of tooth loss and cognitive decline. **(C)** Forest plot of tooth loss and AD.

An association between tooth loss and dementia risk was found in 12 independent studies, with a pooled *RR* of 1.15 (95% *CI*: 1.10–1.20) and moderate heterogeneity (*I*^2^ = 67.4%, *P* < 0.001). Forest plots are shown in Figure 2A.

The pooled *RR* among the Asian population was 1.15 (95% *CI*: 1.10–1.21), but there was no statistical significance from Europe (*RR* = 1.06, 95% *CI*: 0.81–1.38) and North America (*RR* = 1.03, 95% *CI*: 0.94–1.13). The pooled *RR* among mixed-sex population was 1.16 (95% *CI*: 1.09–1.22), while statistical significance was also found among men (*RR* = 1.19, 95% *CI*: 1.09–1.30) and female (*RR* = 1.14, 95% *CI*: 1.01–1.29). The pooled *RR* without the denture use group was 1.70 (95% *CI*: 1.19–2.41), but there was no significant association in the population using dentures (*RR* = 1.04, 95% *CI*: 0.89–1.21). The pooled *RR* among edentulous people and people with natural teeth was 1.24 (95% *CI*: 1.06–1.46) and 1.12 (95% *CI*: 1.06–1.18), respectively. The risk was greater in people with <10 remaining natural teeth (*RR* = 1.22, 95% *CI*: 1.11–1.33) than in those with 20 or more teeth (*RR* = 1.16, 95% *CI*: 1.13–1.20), while it was not significant in those with 10–19 teeth (*RR* = 1.04, 95% *CI*: 0.95–1.13). The pooled *RR* was 1.16 (95% *CI*: 1.10–1.22) in studies in which dental assessment was conducted by a dentist, and 1.12 (95% *CI*: 1.02–1.22) in studies that reported dental status. When dividing studies into ≥5 and <5 years of follow-up, the risk was 1.18 (95% *CI*: 1.12–1.24) and 1.24 (95% *CI*: 1.01–1.51), respectively. For the type of dementia, the pooled *RR* was 1.12 (95% *CI*: 1.02–1.23) for the association between tooth loss and AD (Figure 2C), and 1.25 (95% *CI*: 1.06–1.47) for the association between tooth loss and VaD.

#### 3.3.2. Association between tooth loss and cognitive decline

Of the seven independent studies, the pooled *RR* of cognitive decline was 1.20 (95% *CI*: 1.14–1.26) with moderate heterogeneity (*I*^2^ = 42.3%, *P* = 0.04) (Figure 2B).

The pooled *RR* among populations from Asia, North America and mixed countries was 1.26 (95% *CI*: 1.15–1.38), 1.12 (95% *CI*: 1.05–1.19) and 1.30 (95% *CI*: 1.15–1.46), respectively, but not significant in people from Europe (*RR* = 1.08, 95% *CI*: 0.93–1.27). The pooled *RR* among men and both sexes was 1.10 (95% *CI*: 1.01–1.18) and 1.22 (95% *CI*: 1.16–1.29), respectively. The pooled *RR* among those without dentures was 1.26 (95% *CI*: 1.18–1.34), but no statistical significance was observed among the population using dentures (*RR* = 1.02, 95% *CI*: 0.85–1.22). The pooled *RR* among people with 10–19 teeth and those with fewer than ten teeth was 1.23 (95% *CI*: 1.13–1.33) and 1.20 (95% *CI*: 1.00–1.44), respectively. From the results of the subgroup analysis for edentulous status, the populations without and with natural teeth had a pooled RR of 1.27 (95% *CI*: 1.14–1.41) and 1.20 (95% *CI*: 1.13–1.28), respectively. The pooled risk from studies that used professional dental assessments was 1.16 (95% *CI*: 1.04–1.31), and the risk from those using self-reported questionnaires was 1.17 (95% *CI*: 1.07–1.29). Most (6/8) of the included studies had follow-up for 5 years or more, and the pooled RR was 1.18 (95% *CI*: 1.09–1.27), while the risk was not significant in people who were followed up for <5 years (*RR* = 1.67, 95% *CI*: 0.61–4.53).

### 3.4. Sensitivity analysis

We excluded a single study and pooled the results of the remaining studies. In the sensitivity analysis between tooth loss and dementia risk, none of the individual studies had a significant effect on the pooled *RR*, which ranged from 1.15 (95% *CI*: 1.07–1.23) to 1.20 (95% *CI*: 1.11–1.29) when omitting individual studies individually. In the sensitivity analysis of tooth loss with cognitive decline, the pooled *RR* ranged from 1.17 (95% *CI*: 1.09–1.25) to 1.22 (95% *CI*: 1.11–1.34) when omitting individual studies individually. In summary, the results of the quantitative synthesis based on the sensitivity analysis were stable.

### 3.5. Publication bias assessment

**Figure 3:**
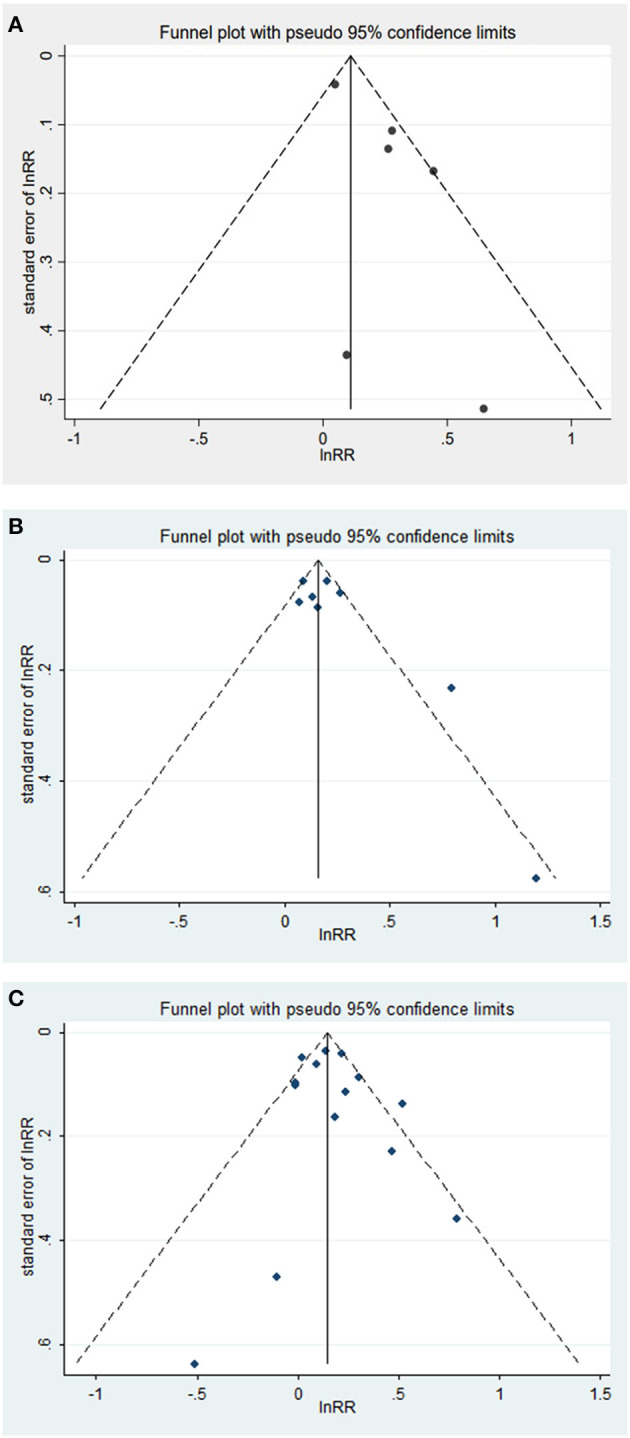
**(A–C)** Funnel plot with pseudo 95% confidence limits between tooth loss and dementia, cognitive decline.

The possibility of publication bias was assessed using a funnel plot. For the association between tooth loss and risk of dementia or cognitive decline, visual inspection of the funnel plots revealed no significant asymmetry (Figure 3). The Begg's and Egger's tests also showed no significant publication bias in the assessment of tooth loss and risk of dementia (*P* from Begg's test = 0.83, *P* from Egger's test = 0.53) or cognitive decline (*P* from Begg's test = 0.39, *P* from Egger's test = 0.11).

## 4. Discussion

The results of this meta-analysis of 21 cohort studies showed that tooth loss was associated with the risk of cognitive decline (*RR* = 1.20; 95% *CI*: 1.14–1.26) and dementia (*RR* = 1.15; 95% *CI*: 1.10–1.20), respectively. These findings suggest that tooth loss is associated with an increased incidence of cognitive decline and dementia. Increased associations were also found between tooth loss and the two most common subtypes of dementia, that is, AD and VaD, with an RR of 1.12 (95% *CI*: 1.02–1.23) and 1.25 (95% *CI*: 1.06–1.47), respectively.

### 4.1. Comparison with previous studies

Our findings showed a positive correlation between tooth loss and dementia incidence (*RR* = 1.15; 95% *CI*: 1.10–1.20), which was not as strong as that from the previous meta-analysis (HR or RR of the included cohort studies between 1.22 and 2.07) (8, 16–19). Compared with these earlier studies, this meta-analysis included more cohort studies and a larger sample size, which significantly increased the statistical power to detect potential associations between tooth loss and dementia risk. The previous meta-analysis by Shen et al. (16) was based on 11 published articles with six cohort studies, three cross-control studies, and two cross-control studies. A previous review performed by Fang et al. (8) included seven cohort studies, eight cross-sectional studies, and two cross-control studies. The reviews by Cerutti-Kopplin et al. (17), Chen et al. (18), and Oh et al. (19) only included 8, 8, and 11 cohort studies, respectively. However, our review included 21 cohort studies with a large number of sample size (a total of 356,297) with a long follow-up period (8.6 years on average), which could decrease the sampling error to a great extent. Moreover, apart from dementia, our study also explored the relationship between tooth loss and the risk of cognitive decline and two common subtypes of dementia, AD and VaD. Additionally, the current meta-analysis investigated a more detailed subgroup analysis, including area, sex, dementia type, use of dentures, number of teeth and edentulous status, dental assessment methods, and follow-up duration of the associations between tooth loss and various cognitive disorders, including all types of dementia, cognitive decline, and dementia due to AD (see Table 1 and Supplementary Table 3).

**Table 1:** Subgroup analysis of relative risks for the association between tooth loss and both dementia and cognitive decline.

**Subgroup**		**No of studies**	**Relative risk**	**95%*CI***	**Heterogeneity**		
					***Q***	***P***	***I*^2^ (%)**
**Subgroup analysis of dementia**							
Overall			1.15	1.10–1.20	117	<0.01	67.4
Geographic location	Asia	7	1.15	1.10–1.21	132	<0.01	79.6
	Europe	3	1.06	0.81–1.38	10	0.11	42.3
	North America	3	1.01	0.92–1.12	9	0.57	0.0
	Mixed	1	1.35	1.14–1.60	2	0.16	50.2
Sex	Male	3	1.19	1.09–1.30	2	0.89	0.0
	Female	5	1.14	1.01–1.29	17	0.049	47.0
	Mixed	9	1.16	1.10–1.22	101	<0.01	74.2
Denture use	No	2	1.70	1.19–2.41	0	0.71	0.0
	Yes	3	1.04	0.89–1.21	1	0.87	0.0
	Mixed	12	1.15	1.09–1.20	110	<0.01	70.8
Edentulousness	No	9	1.12	1.06–1.18	81	<0.01	72.9
	Yes	6	1.15	1.10–1.20	11	0.13	37.8
	Mixed	7	1.21	1.07–1.36	20	0.01	59.3
Number of teeth	0–9	9	1.22	1.11–1.33	36	<0.01	63.7
	10–19	3	1.04	0.95–1.13	9	0.06	56.0
	≥20	2	1.16	1.13–1.20	0	0.53	0.0
	Mixed	9	1.12	1.02–1.22	35	<0.01	59.4
	Unspecific	2	1.17	1.06–1.28	2	0.34	6.3
Tooth loss assessment	By a dentist	9	1.16	1.10–1.22	93	<0.01	74.1
	Self-report	5	1.12	1.02–1.22	23	0.04	43.1
Follow-up duration	≥5 years	11	1.18	1.12–1.24	57	<0.01	52.7
	< 5 years	2	1.24	1.01–1.51	5	0.32	15.5
	Mixed	1	1.08	1.00–1.17	34	<0.01	85.2
Type	AD	6	1.12	1.02–1.23	15	0.053	47.9
	VaD	3	1.25	1.06–1.47	18	<0.01	71.5
	Mixed	13	1.17	1.10–1.23	93	<0.01	64.5
**Subgroup analysis of cognitive decline**							
Overall			1.20	1.14–1.26	26	0.04	42.3
Geographic location	Asia	3	1.26	1.04–1.53	29	0.18	45.2
	Europe	1	1.08	0.93–1.27	0	0.82	0.0
	North America	3	1.12	1.05–1.19	1	0.88	0.0
	Mixed	1	1.30	1.15–1.46	2	0.18	45.2
Sex	Male	1	1.10	1.01–1.18	0	0.84	0.0
	Mixed	7	1.22	1.16–1.29	20	0.10	34.4
Denture use	No	2	1.26	1.18–1.34	5	0.30	18.4
	Yes	2	1.02	0.85–1.22	16	<0.01	74.9
	Mixed	6	1.17	1.09–1.25	14	0.08	42.9
Edentulousness	No	2	1.20	1.13–1.28	5	0.31	16.2
	Yes	3	1.27	1.14–1.41	8	0.18	37.3
	Mixed	4	1.12	1.05–1.20	4	0.38	5.4
Tooth number	0–9	5	1.20	1.00–1.44	32	<0.01	78.4
	10–19	1	1.23	1.13–1.33	0	–	–
	Mixed	3	1.23	1.12–1.35	3	0.39	0.2
	Unspecific	2	1.11	1.04–1.18	0	0.86	0.0
Tooth loss assessment	By a dentist	4	1.16	1.04–1.31	11	0.15	35.3
	Self-report	4	1.17	1.07–1.29	27	<0.01	74.4
Follow-up duration	≥5 years	6	1.18	1.09–1.27	38	<0.01	66.0
	< 5 years	2	1.67	0.61–4.53	3	0.07	70.5

More importantly, some valuable findings were observed. Our meta-analysis showed a significant association between an increased risk of dementia and cognitive decline in Asian populations with tooth loss, whereas the risks were lower or not statistically significant in people from Europe and North America with missing teeth. The World Health Organization's Global Burden of Disease Study 2019 (GBD 2019) anticipated an increasing trend in dementia prevalence due to population growth and aging, and found wide geographic heterogeneity in the projected increase in dementia cases across countries and regions (45). However, the present studies have been conducted in only a few countries and regions. Few studies were conducted in Africa and the Middle East, where the number of predicted dementia cases was greatest. For both dementia and cognitive decline, the risk of tooth loss was higher in people who did not wear dentures than in those with dentures. In subgroup analysis of the number of remaining natural teeth, the association between tooth loss and risk of dementia, cognitive decline, or AD was higher in the edentulous population than in the population with natural teeth, while higher AD and risk of all types of dementia were observed in people with only nine or fewer teeth than in people with 10–19 teeth. The results suggested a dose-response relationship between the number of natural teeth and the risk of dementia risk; however, further dose-response analyses are needed. In a quick summary, Daly et al. (46) noted that current epidemiologic research is generally based on self-report measures of oral health. Considering the low knowledge and awareness of oral health in the public, this would lead to a considerable risk of error and heterogeneity across studies.

Dementia is a chronic disease and has a longer prodromal period than other diseases. However, there is no firm evidence on the duration from exposure to tooth loss to the onset of dementia/cognitive decline, and there are no guidelines or instructions for a standard follow-up duration for research on this topic. The World Health Organization's Global Burden of Disease Study 2019 showed that the prevalence of dementia is increasing “doubling about every 5 years until 85 years of age in both 2019 and 2050” (45), so we divided the follow-up duration into <5 and ≥5 years. Most of the included studies (17/21) had a follow-up of more than 5 years, and their pooled *RRs* for all outcomes had narrow CI, allowing the results to provide precise estimates of population values. Some studies suggested a follow-up of 10 years (47, 48), so these risk factors in earlier life could be identified, patients in earlier stages of dementia be diagnosed, and reverse causation be minimized, especially in studies, except prospective cohorts. In our meta-analysis, more than half (11/21) of the included studies were followed for <10 years or more (at least 2 years). Future research should investigate the causality with a sufficiently long follow-up period.

Dementia is a disease that results in diminishing language abilities, judgement, memory loss, and behavioral abnormalities due to damage to cerebral neurons caused by progressive, degenerative, and cerebrovascular disease (49, 50). Although recent research suggests a link between poor dental health and accelerated dementia and cognitive decline, there are currently no clear treatments for dementia, and the mechanism by which cognitive impairment is associated with tooth loss remains unknown. Therefore, it is important to identify modifiable risk factors that support dementia prevention.

Several possible mechanisms have been proposed to explain the effects of tooth loss on cognitive function (51–58). Nutrition, inflammation, and neural feedback are the most commonly suggested factors (59). Theories of nutrition and neural feedback are related to masticatory dysfunction caused by tooth loss. Tooth loss means decreased chewing function, which leads to poorer digestive capacity and appetite, and then decreased nutrition, which ultimately damages brain functions such as cognition. Deficiency of several nutrients have been found to be related to the risk of cognitive decline and dementia, like vitamin D deficiency (60, 61) and iron deficiency (62). While higher intake of some nutrients and dietary interventions also seems to be associated with lower risk of cognitive dysfunction (63, 64). The causally link of vitamin D deficiency to dementia was observed, even after excluding the influence of genetic factors by using Mendelian randomization study design (65, 66). The metabolic, endocrine, immune functions and neuronal effects of 1 alpha, 25-dihydroxyvitamin D (1, 25(OH)2D) would be considered an essential contributor to the development of cognitive disorders (67, 68). Tooth loss (10–15) and vitamin D deficiency (61, 69–71) are both found to be related to many systemic health problems, and which further indicates an important role of low 1, 25(OH)2D concentration in the relationship between tooth loss and systemic diseases including cognitive damage. Low-oxygen supply (72) and brain iron dyshomeostasis (73) may explain the iron-related neuropathogenesis of cognitive impairment or dementia. This may also partly explain why evidence-based guidelines recommend dietary interventions to reduce the risk of cognitive decline and dementia (59). However, few interventional studies have proved causality between the deficiency of vitamin D and iron and the risk of dementia. Morever, attention should also be paid to the likely of prolonged supplementation or overload of vitamin D and iron (73, 74).

Besides, fewer interocclusal contacts related to decreased masticatory function indicate impaired proprioception and less somatosensory feedback, and such changes would lead to cognitive decline and dementia (75). Studies in animals and humans have shown the effects of dysfunction of the masticatory apparatus on cognitive damage and the reverse effect of masticatory activities on cognitive processes, indicating preventive and curative effects of chewing on cognitive performance (76–79). The number of teeth remaining rather than lost, e.g., 16, 20, or whatever, was used for the classification because they were thought to be sufficient for mastication. Number 20 without a third molar is usually recommended as the cut-off (23, 28, 34, 39, 41), since it is at least one posterior occlusal unit, the most important factor affecting a person's ability to chew (80). In fact, the number of lost teeth were assessed only in four out of the 18 involved studies (22, 29, 32, 33) in our meta-analysis. A 2018 Australian cross-sectional study found that the number of teeth (<20) was associated with a lower ability to chew hard foods and discomfort with eating, both of which were related to cognitive status, suggesting a chain of tooth loss, chewing ability, and cognitive damage (81). A cross-national study of three large cohorts in Europe and New Zealand confirmed in 2019 that lack of intact teeth and difficulty chewing are predictive of poor cognition and three other aspects of overall health in older people (82). However, there are some controversial results on whether tooth loss explains the association between cognition and mastication. Lexomboon et al. (83) conducted a study based on a nationally representative sample of an elderly Swedish population and reported that the likelihood of cognitive impairment, adjusted for sex, age, and education, was not significantly different between elderly people with and without multiple tooth loss, but remained significantly higher in those with difficulty chewing. The study suggests that difficulty chewing, not tooth loss, is an independent risk factor for cognitive impairment. Further evidence from studies based on a cohort design across different geographic areas and with adequate follow-up time is needed to confirm the relationship between tooth loss, chewing ability, and cognition.

Inflammation is one of the most commonly proposed theory to explain the effects of tooth loss on cognitive function. The theory is that without the protection of the teeth that results from tooth loss, the gingival tissue cannot protect the central nervous system (CNS) from constant exposure to pathogenic oral bacteria that can lead to or exacerbate inflammatory processes, causing endovascular inflammatory mediators and neurodegeneration. Thus, cognitive deficits and dementia may precede inflammation in the CNS (30, 84, 85). Periodontitis may also be related to cardiovascular and cerebrovascular disease, which are associated with the onset of dementia (10, 53, 86). This inflammation theory is also supported by epidemiological studies, which report a higher risk and severity (87, 88), clinical studies that observed bacterial evidence in patients with dementia and periodontitis (89), and animal experiments (90). Excessive nitric oxide levels (NO) are associated with excessive cell death and inflammation. Pang et al. (90) compared the effects of tooth loss with those of chronic cerebral ischaemia on cognitive function in Wistar rats and found that the effects were similar; NO, inducible nitric oxide (iNOS), and endothelial nitric oxide synthase (eNOS) in the hippocampus were involved in both cases. Some of the involved studies in our meta-analysis also paid attention to the problem of periodontitis and dental caries and found an increased risk for cognitive damage (22, 23). However, few studies have examined the relationship between the incidence of dementia and the number of teeth lost after accounting for other dental problems and/or behaviors, such as chewing difficulties and periodontitis (22, 41). It would be prudent for future studies to consider potential confounding or interactions with other dental problems to demonstrate an independent causative role of tooth loss on the risk cognitive impairment.

Further, poor cognitive status may also precede tooth loss. People having dementia are usually observed to have poor oral conditions and tooth loss, even in the early stage of the disease (91). Foley et al. (92) have conducted and demonstrated the effect of tooth loss on cognitive status. It may be caused by impaired cognitive function, memory, and physical ability. The risk of tooth loss resulting from poor cognitive function should also be paid attention to when discussing the relationship between tooth loss and cognitive impairment, i.e., the bi-direction association between them.

### 4.2. Strengths and limitations

The strength of our study is that it includes twice as many studies as previous reviews, with a large sample size of up to 35 thousand and an average follow-up time of 8 years. This makes our findings more reliable and generalisable. Additionally, this study demonstrates associations between tooth loss and various cognitive disorders, including subclinical cognitive decline, AD, VaD, and dementia of all types. Finally, we conducted a series of subgroup analyses focusing on the baseline characteristics of the subjects (sex and region), study design (follow-up period and dental assessment method), and type of cause and outcome, which could provide a wealth of insights for research on the relationship between dental health and cognitive status. The possible confounding effects of age on the relationship between tooth loss and cognitive decline had been also dealt with, as age was used as a covariate in most of the included studies (19/21, see Supplementary Table 1 “Characteristics of included studies in the meta-analysis”).

In interpreting this meta-analysis, the following limitations must be considered: first, the included studies used different methods and criteria to measure tooth loss and outcomes. For example, the number of teeth in some studies was self-reported by participants and not confirmed by a dentist or dental records, which could affect the accuracy of our results. Although we attempted to pool adjusted risk estimates, adjusted confounders were not available or varied widely among included studies. Although the mechanisms and risk factors of dementia and cognitive decline are far from clear, the incidence of dementia increases with age and varies between men and women. The evidence supporting 12 modifiable dementia risk factors was emphasized by the Lancet Commission on Dementia Prevention, Intervention, and Care (93). However, some of the above 12 potentially important confounders, such as sex, education, diet, smoking, hypertension, depression, dental health behaviors, and status, were neither fully adjusted for nor used as stratifying factors in some of the included studies, which may have influenced the assessment of the results. Third, the time of occurrence of tooth loss and the length of the edentulous period cannot be precisely determined, which may introduce bias in the incomparability of results to some extent (19).

## 5. Conclusions

This meta-analysis suggests that tooth loss is associated with increased risk of cognitive decline and dementia, as well as the two most common types of dementia, AD and VaD. Underlying factors that may be the cause of the observed associations include nutrition, inflammation, and neural feedback, especially deficiency of several nutrients like vitamin D. The results of our study suggest the importance of dental care in maintaining teeth adequately and protecting against cognitive impairment.

## Data availability statement

The original contributions presented in the study are included in the article/Supplementary material, further inquiries can be directed to the corresponding author.

## Author contributions

Article evaluation: LL, CW, QZ, YZ, and DY. Data extraction: CW, SY, and DY. Data analysis: CW, DY, SY, and YZ. Results interpretation: MJ, XW, LZ, and QL. Drafting the article: LL and CW. Critical revision of the manuscript: CW, LL, QZ, DY, SY, ZL, YG, and XZ. Final approval of the manuscript: all the authors. They have also agreed both to be personally accountable for their own contributions and to ensure that questions related to the accuracy or integrity of any part of the work, even ones in which the author was not personally involved, are appropriately investigated, resolved, and the resolution documented in the literature.
