# Supplementary material for: Tooth loss and the risk of cognitive decline and dementia: A meta-analysis of cohort studies
Source: Front Neurol. 2023 Apr 17;14:1103052. doi: 10.3389/fneur.2023.1103052 (PMC10150074; doi:10.3389/fneur.2023.1103052)
Supplement: Supplementary file 1 [file Table_1.pdf]

Supplemental Table 1 Characteristics of included studies in the meta-analysis

Study source	Sex	Sample	Study name	Country	Type	Baseline age d(years)	Tooth loss assessment	Outcome assessment	Follow-up time (years)	Covariates
Shimazaki et al. 2001	M/F	517	None	Japan	Mental Impairment	79.7 ± 7.5 (range:59 -107)	Dental examination	Medical records	6	Age, physical health status, classification of institution and cerebrovascular disorder
Kim et al. 2007	M/F	686	Dementia in Developing Countries Research Program	South Korea	AD,VaD and other subtypes of dementia	mean 73.4	Dental examination	Cognitive examinations	2	Age, sex and education, vascular disease/risk (Stroke, heart disease, hypertension, diabetes and smoking status)
Stein et al. 2007	M/F	133	Nun Study	USA	Dementia	75-98 (mean 84 yrs)	Medical records	Cognitive examinations	12	Age, education and apolipoprotein E4 allele
Kaye et al. 2010	M	597	Veterans Affairs Dental Longitudinal Study	USA	Poor Cognitive Function	28-70	Dental examination	Cognitive examinations	10	Coronary heart disease, average alcohol intake (g/day), and number of teeth at baseline
Yamamoto et al. 2012	M/F	4425	Aichi Gerontological Evaluation Study	Japan	Dementia		Self-reported questionnaire	Insurance records	4	Age, household income, BMI, present illness, alcohol consumption, exercise, and forgetfulness

			(AGES)							
Arrive´ E et al. 2012	M/F	405	Project Personnes Age´es QUID (PAQUID) Study	France	AD, VaD, and other subtypes of dementia	>65	Dental examinations	Neuropsychological testing	10	Sex, BMI, diabetes, depression, hypertension and ischemic cardiopathy/history of brain stroke
Paganini-Hill et al. 2012	M/F	5468	Leisure World Cohort Study	USA	Dementia	52-105 (median 81)	Self-reported questionnaire	questionnaires, hospital records, death certificates, and in-person neurological and neuropsychological evaluations	12	Age at entry, smoking, alcohol, caffeine, active activities, other activities, BMI, high blood pressure, angina pectoris, heart attack, stroke, diabetes mellitus, rheumatoid arthritis, cancer, education, head trauma, and family history of dementia.
Reyes-Ortiz et al. 2013	M/F	1967	Hispanic Established Populations for Epidemiologic Studies of the Elderly (EPESE)	American	Cognitive impairment	65+	In-home interview	In-home interview (MMSE)	5	None

Batty et al. 2013	M/F	11140	Action in Diabetes and Vascular disease: Preterax and Diamicron Modified-Release Controlled Evaluation (ADVANCE) trial	20 countries (in Australasia, Asia, Europe and North America)	Dementia and cognitive decline	55-88 (mean 65.78) yrs	Questionnaire	Professional-administered questionnaire (MMSE)and specialist diagnosis	5	Age, sex, existing illness (one or more of the following: use of metformin/beta-blockers, history of macrovascular or microvascular disease, or those requiring assistance with daily activities, plus diabetes duration), behavioural CVD risk factors (cigarette smoking, alcohol intake, vigorous physical activity in previous week),physiological CVD risk factors (Haemoglobin A1c, creatinine, BMI, total cholesterol, HDL cholesterol, resting heart rate, SBP, DBP), psychological CVD risk factors (Quality of life (EQ-5d score), Socio-economic CVD risk factors (age at completion of highest level of education, height), treatment allocation and ethnicity
Hansson et al. 2014	M/F	1408	Betula prospective cohort study	Sweden	Dementia	71.2±8.2	Self-reported questionnaire	Psychiatrist diagnosis	20	Age, sex, formal education, apolipoprotein E-4 status

Stewart etal. 2015	F	580	Prospective Population Study of Women (PPSW)	Sweden	AD, VaD, and other types of dementia	77.09±5.64	Clinical examination and radiological survey	Neuropsychiatric examinations and medical records	13	Age
Takeuchi et al. 2017	M/F	1566	Hisayama Study	Japan	All-cause dementia	60+	Oral examination	Neurological examinations, neuropsychological tests, interviews and medical records	5	Sex, age, occupation, education, hypertension, diabetes mellitus, history of stroke, alcohol intake, tooth brushing frequency, regular visits to the dentist, and denture use
Chen et al. 2010	M/F	134	None	USA	Dementia	73.8± 10.7 (44-103)	Medical records	Medical records	7	None
Stewart et al. 2013	M/F	945	Health, Aging and Body Composition (Health ABC) Study	USA	Cognitive Decline	73.5 ± 2.8	Visual inspection	Cognitive assessment (3MS)	3	None
Tsakos et al. 2015	M/F	3166	the English Longitudinal Study of	England	Poor memory	70.57± 2.18	Self-reported questionnaire	Cognitive assessment (10-word	10	Time, age, sex, marital status, socioeconomic position (education and wealth),

			Ageing (ELSA)					recall test)		number of cardiovascular and noncardiovascular comorbidities, gait speed category, health behaviors (smoking, frequency of alcohol consumption, physical activity), depressive symptoms, BMI, and waist circumference
Saito et al. 2018	M/F	491	Ohasama study	Japan	Cognitive impairment	70.9 ± 4.3	Dental examination	Cognitive assessment (MMSE)	4	Age, sex, hypertension, diabetes, cerebrovascular/cardiovascular disease, hypercholesterolemia, depressive symptoms, BMI, current smoker status, current drinker status, duration of education, and baseline MMSE score
Yoo et al. 2019	M/F	209806	National Health Insurance Service-Elderly Cohort Database (NHIS-ECD)	South Korea	Dementia	60-103 (mean 67.5)	Medical records	Medical records	11	Age, sex, hypertension, diabetes, cerebrovascular/cardiovascular disease, hypercholesterolemia, depressive symptoms, BMI, current smoker status, current drinker status, duration of education, and baseline

									MMSE score
Kim et al. 2020	M/F	20230	National Health Insurance Service-National Health Screening Retrospective Cohort (NHIS-HEALS)	South Korea	AD, VaD, and comorbid of AD and VaD	78.1±4.9	Dental examination	Diagnosed By a neurologist and psychiatrist	13	Sociodemographic, anthropomorphic, and lifestyle factors (sex, age, household income, insurance status, BMI, total cholesterol, smoking and drinking status, and frequency of physical activity) and comorbidities (hypertension and diabetes mellitus).
Kim et al. 2021	M/F	39810	Korean National Health Insurance Service Health Screening Cohort study	Korea	AD	⩾60	Dental examination	ICD-10, ICD-10-CM	13	obesity, smoking, alcohol consumption, systolic blood pressure, diastolic blood pressure, fasting blood glucose, total cholesterol, and the Charlson Comorbidity Index scores
Kiuchi et al. 2022	M/F	35744	Japan Gerontological Evaluation Study	Japan	Dementia	73.2 (73.1 ± 5.5 for men and 73.2 ± 5.5 for women)	Self-reported questionnaire	Dementia Scale in the Japanese Long-term Care Insurance	6	weight loss, vegetable and fruit intake, homeboundness, social network as mediators; age and baseline, marital status, denture use, income, education level, hypertension,

								System		diabetes mellitus, drinking history, smoking history, and walking time included as covariates
Yang et al. 2022	M/F	17079	Chinese Longitudinal Healthy Longevity Survey	China	Cognitive impairment	median 83 years, IQR 74-90 years	Self-reported questionnaire	MMSE	median 9.1 years (IQR 3.8–13.3 years)	age, sex, ethnicity, education level, residence, coresidence, sufficient income, smoking, drinking, fruit intake, vegetable intake, body mass index, hypertension, activities of daily living, self-rated health

Abbreviation: M, male; F, female; AD, Alzheimer's disease; VaD, vascular dementia; MMSE, Mini-mental State Examination; 3MS, Modified Mini-Mental State Examination; CVD, cardiovascular disease; BMI, Body Mass Index; SBP, systolic blood pressure; DBP, diastolic blood pressure; HbA1c, Glycated haemoglobin; ICD, International Classification of Diseases; CM, Clinical Modification; IQR, interquartile range.
