# Supplementary material for: Tooth loss and the risk of cognitive decline and dementia: A meta-analysis of cohort studies
Source: Front Neurol. 2023 Apr 17;14:1103052. doi: 10.3389/fneur.2023.1103052 (PMC10150074; doi:10.3389/fneur.2023.1103052)
Supplement: Supplementary file 2 [file Table_2.pdf]

Supplementary Table 2 Quality assessment of studies

Original study	Selection				Comparability	Exposure			Total score
	Representativeness of the exposed cohort	Selection of the non-exposed cohort	Ascertainment of exposure	Demonstration that outcome of interest was not present at start of study	Comparability of cohorts on the basis of the design or analysis	Assessment of outcome	Was follow-up long enough for outcomes to occur	Adequacy of follow up of cohorts	
Shimazaki et al. 2001	1	1	1	1	2	1	1	1	9
Kim et al. 2007	1	1	1	1	2	1	1	1	9
Stein et al. 2007	0	1	1	1	2	1	1	1	8
Kaye et al. 2010	0	1	1	1	1	1	1	1	7
Yamamoto et al. 2012	1	1	0	1	2	1	1	1	8
Arrive´ et al. 2012	0	1	1	1	1	1	1	1	7
Paganini-Hill et al. 2012	1	1	0	1	2	1	1	1	8
Reyes-Ortiz et al. 2013	1	1	1	1	0	1	1	0	6
Batty et al. 2013	1	1	0	1	2	1	1	1	8
Hansson et al. 2014	0	1	0	1	2	1	1	1	7
Stewart et al. 2015	1	1	1	1	1	1	1	1	8
Takeuchi et al. 2017	1	1	0	1	2	1	1	1	8
Chen et al. 2010	0	1	1	1	0	1	1	0	5
Stewart et al. 2013	0	1	1	1	0	1	1	1	6
Tsakos et al. 2015	1	1	1	1	2	1	1	1	9
Saito et al. 2018	1	1	1	1	2	1	1	1	9
Yoo et al. 2019	1	1	1	1	2	1	1	1	9
Kim et al. 2020	1	1	1	1	1	1	1	0	7
Kim et al. 2021	1	1	1	1	2	1	1	1	9
Yang et al. 2022	1	1	1	1	2	1	1	0	8
Kiuchi et al. 2022	1	1	0	1	2	1	1	0	7
