# Supplementary material for: Tooth loss and the risk of cognitive decline and dementia: A meta-analysis of cohort studies
Source: Front Neurol. 2023 Apr 17;14:1103052. doi: 10.3389/fneur.2023.1103052 (PMC10150074; doi:10.3389/fneur.2023.1103052)
Supplement: Supplementary file 3 [file Table_3.pdf]

Supplemental Table 3 Subgroup analysis of relative risks for the association between tooth loss and AD

Subgroup		No of studies	Relative risk	95%*CI*	Heterogeneity		
					Q	*P*	I^2 (%)
Overall			1.11	1.03-1.21	28	<0.01	63.9
Location	Asia	4	1.11	1.02-1.20	26	<0.01	69.7
	Europe	1	1.91	0.70-5.23	0	-	-
	North America	1	1.10	0.47-2.58	0	-	-
Sex	Male	1	1.28	1.11-1.49	0	0.68	0.0
	Female	3	1.19	1.12-1.27	1	0.83	0.0
	Mixed	3	1.09	1.00-1.18	23	<0.01	68.9
Edentulousness	No	2	1.06	0.98-1.16	18	<0.01	72.9
	Yes	1	1.62	0.87-3.03	0	-	-
	Mixed	4	1.32	1.12-1.55	1	0.87	0.0
Tooth number	<9	4	1.13	1.03-1.25	10	0.08	49.0
	10-19	2	0.99	0.91-1.08	2	0.29	19.4
	Unspecific	2	1.31	1.11-1.55	0	0.93	0.0
Follow-up duration	≥ 5 years	4	1.39	1.17-1.65	2	0.89	0.0
	< 5 years	1	1.30	1.00-1.70	0	-	-
	Mixed	1	1.05	0.96-1.13	15	<0.01	79.3
